# Supplementary figures and images for: Post-Harvest Application of Nanoparticles of Titanium Dioxide (NPs-TiO2) and Ethylene to Improve the Coloration of Detached Apple Fruit
Source: Foods. 2023 Aug 21;12(16):3137. doi: 10.3390/foods12163137 (PMC10453011; doi:10.3390/foods12163137)

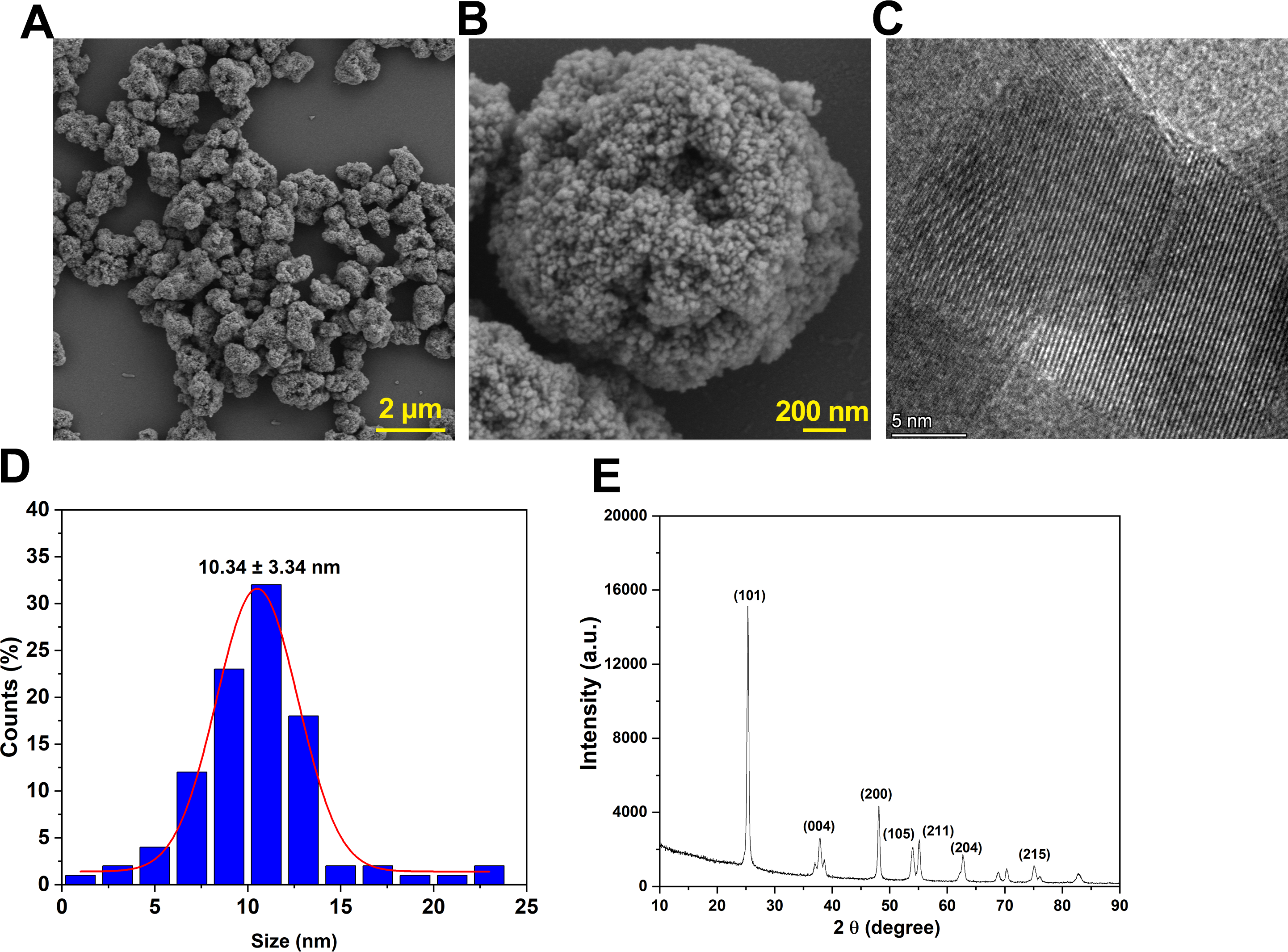

Supplement: Supplementary file 1 [file foods-12-03137-s001.zip › supplementary Figure S1.tif]

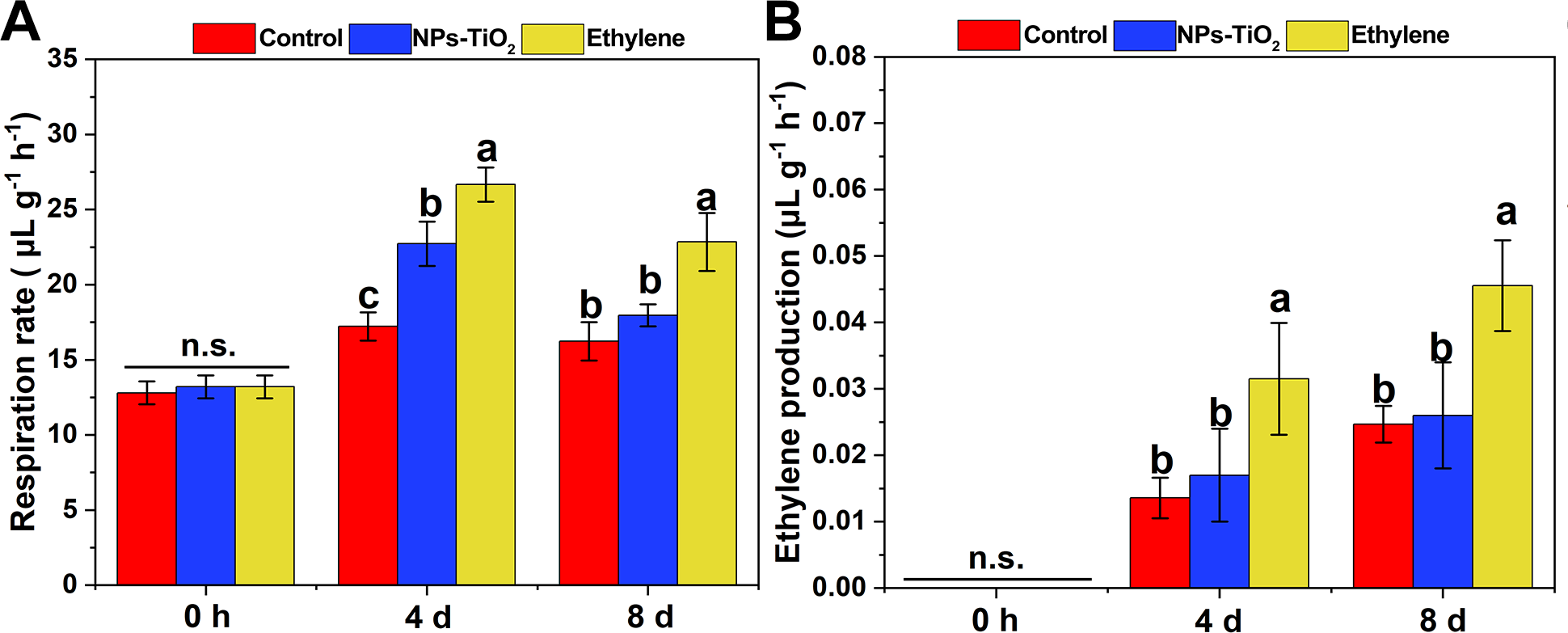

Supplement: Supplementary file 1 [file foods-12-03137-s001.zip › supplementary Figure S2.tif]

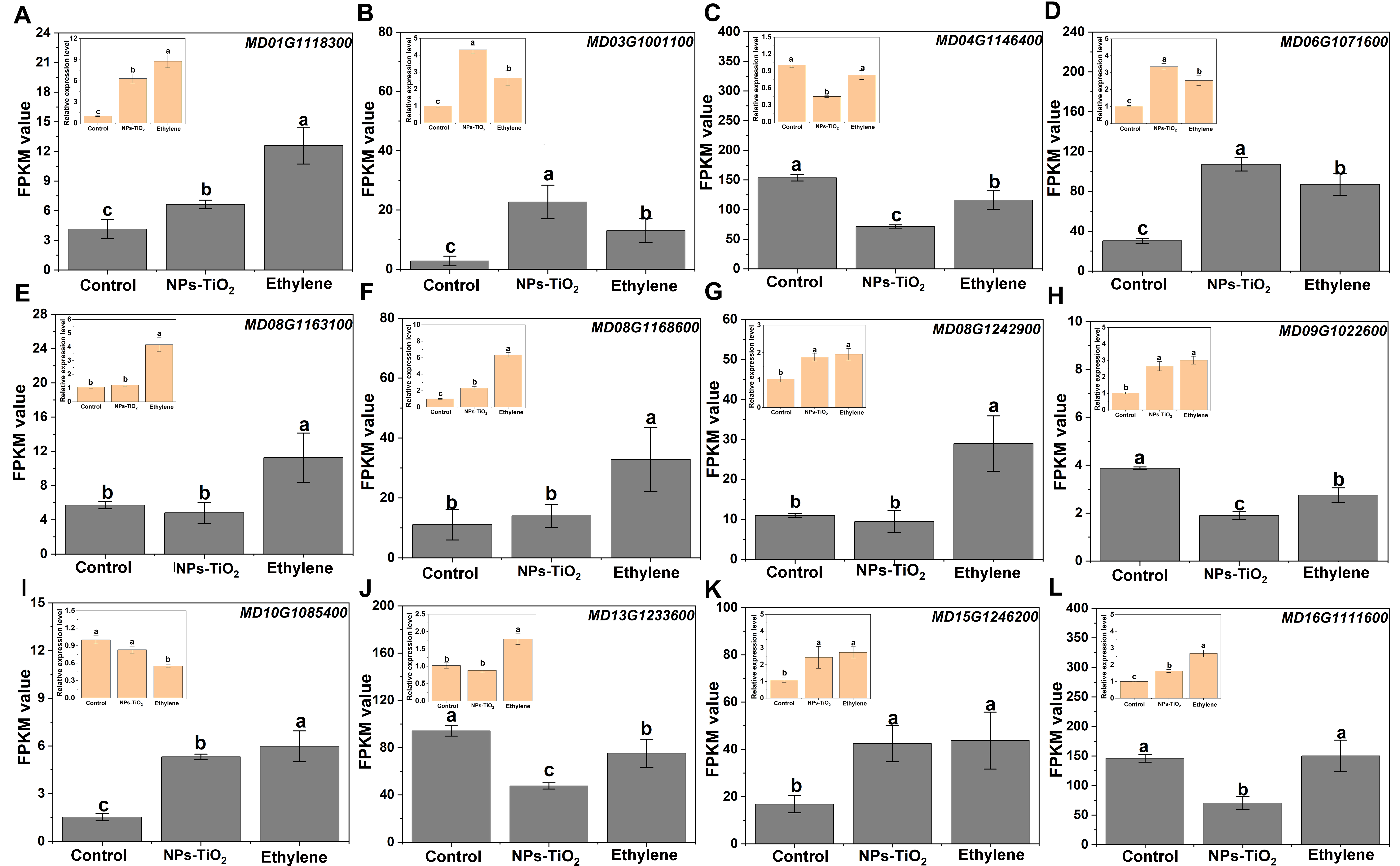

Supplement: Supplementary file 1 [file foods-12-03137-s001.zip › supplementary Figure S3.tif]

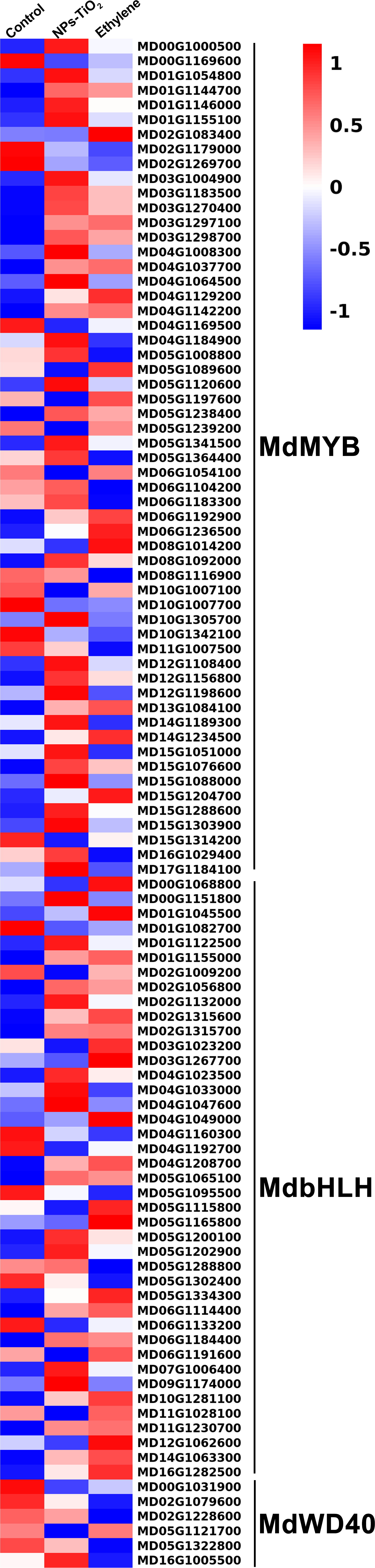

Supplement: Supplementary file 1 [file foods-12-03137-s001.zip › supplementary Figure S4.tif]
